# Efficacy and safety of secondary induction radiotherapy in locally advanced resectable esophageal squamous cell carcinoma with poor responses to neoadjuvant immunochemotherapy: a retrospective study

**DOI:** 10.3389/fimmu.2026.1656631

**Published:** 2026-03-11

**Authors:** Huilai Lv, Ping Zhang, Liang Dai, Weilu Ding, Songping Xie, Hongjing Jiang, Xiaofeng Duan, Ke-Neng Chen, Ziqiang Tian

**Affiliations:** 1 Department of Thoracic Surgery, The Fourth Hospital of Hebei Medical University, Shijiazhuang, Hebei, China; 2 Hebei Key Laboratory of Accurate Diagnosis and Comprehensive Treatment of Esophageal Cancer, Shijiazhuang, Hebei, China; 3 Department of Radiation Oncology, The Fourth Hospital of Hebei Medical University, Shijiazhuang, Hebei, China; 4 Key Laboratory of Carcinogenesis and Translational Research (Ministry of Education), Peking University School of Oncology, Beijing, China; 5 The First Department of Thoracic Surgery, Peking University Cancer Hospital and Institute, Beijing, China; 6 Department of Thoracic Surgery, Renmin Hospital of Wuhan University, Wuhan, China; 7 Department of Minimally Invasive Esophageal Surgery, Tianjin Medical University Cancer Institute and Hospital, National Clinical Research Center for Cancer, Tianjin, China; 8 Key Laboratory of Cancer Prevention and Therapy, Tianjin’s Clinical Research Center for Cancer, Tianjin, China

**Keywords:** esophageal squamous cell carcinoma, neoadjuvant immunochemotherapy, R0 resection, retrospective study, secondary induction radiotherapy

## Abstract

**Purpose:**

Multiple prospective trials have validated the efficacy and safety of neoadjuvant immunochemotherapy (nICT) in resectable esophageal squamous cell carcinoma (ESCC), yet patients with stable disease (SD) or progressive disease (PD) following nICT have limited benefit and are at risk for suboptimal surgical and pathological outcomes. The secondary induction radiotherapy prior surgery may represent a potential therapeutic strategy in this setting.

**Methods:**

We conducted a retrospective study involving 23 patients with locally advanced, resectable ESCC who demonstrated poor response to nICT (19 with SD and 4 with PD). All patients subsequently received secondary induction radiotherapy via intensity-modulated techniques or tomotherapy (median dose, 41.4 Gy). The primary endpoint was the rate of R0 resection. The secondary endpoints included pathological complete response (pCR), major pathological response (MPR), the objective response rate (ORR), the disease control rate (DCR), tumor downstaging, and treatment-related adverse events (TREAs).

**Results:**

Among the 23 patients (median age, 63 years; 82.6% male), 30.4%, 60.9%, and 8.7% had clinical stages II, III, and IVa disease, respectively. Following secondary induction radiotherapy, 4 patients (17.4%) achieved a complete response (CR), and 8 (34.8%) achieved a partial response, yielding an ORR of 52.2% and a DCR of 100%. All patients underwent resection, with an R0 resection rate of 100%. MPR was achieved in 43.5% of patients, and pCR was achieved in 21.7%. Clinical downstaging occurred in 60.9% of patients, and pathological downstaging occurred in 69.6%. The median interval from the end of radiotherapy to surgery was 48 days. Grade 3–4 surgical complications occurred in 8.7% of the patients. Radiation-related grade 3–4 adverse events included myelosuppression in 13.0% and gastrointestinal symptoms in 8.7% of the patients.

**Conclusions:**

In patients with resectable ESCC and a poor response to nICT, secondary induction radiotherapy was associated with encouraging surgical and pathological outcomes and an acceptable safety profile. Prospective studies are warranted to confirm these findings.

## Introduction

Esophageal cancer remains a major global health concern, with an estimated 604,100 new cases and 544,076 deaths reported in 2020 (1). More than half of these cases occur in China, where esophageal squamous cell carcinoma (ESCC) is the predominant histologic subtype, in contrast to the predominance of esophageal adenocarcinoma in Western countries (2). The prognosis of ESCC remains poor, largely due to late-stage diagnosis stemming from nonspecific symptoms and limitations in early detection strategies (3).

Surgical resection is a mainstay of treatment for locally advanced ESCC, offering a 5-year overall survival rate of 52.9% (4). However, postoperative recurrence occurs in approximately 33.7% of patients, underscoring the need for improved preoperative strategies (4). The addition of immunotherapy to chemotherapy has shown synergistic antitumor effects across multiple cancer types (5–7), enhancing tumor immunogenicity and modulating immune responses through the activation of cytotoxic T cells and depletion of immunosuppressive cell populations (8–11). Recent important studies have explored cellular and molecular factors affecting immunotherapy. For example, glycolysis-driven histone lactylation in breast cancer is closely linked to tumor immune responses and immune-related pathway regulation (12). Solid tumors exhibit multiple subtypes due to cellular and molecular heterogeneity, with immune cell infiltration in the tumor microenvironment correlating with immunotherapy efficacy (13). Additionally, the stemness index is a potential biomarker for cancer progression and prognosis; studies on non-small cell lung cancer showed that patients with a high stemness index responded better to PD-1/PD-L1 blockade immunotherapy (14).

Recent phase II and III trials evaluating neoadjuvant immunochemotherapy (nICT) in locally advanced ESCC patients have demonstrated favorable surgical outcomes and manageable toxicity profiles (15–18). Despite these advances, a subset of patients remains ineligible for surgery following nICT due to inadequate tumor regression or disease progression (19, 20). Data from prospective trials suggest that up to 25% of patients fail to undergo surgical resection after completing nICT. For example, in the ESONICT-2 study, only 15 of 20 patients (75.0%) underwent surgery after receiving toripalimab in combination with chemotherapy (21).

Radiotherapy may increase resectability by reducing the tumor burden, decreasing viability, and facilitating clearer intraoperative margins through induced fibrosis at the tumor–normal tissue interface (22–25). On the basis of these observations, we hypothesized that secondary induction radiotherapy could improve the clinical response and surgical outcomes in patients with a poor response to nICT.

## Methods

### Study design and patients

Patients aged ≥18 years with histologically confirmed ESCC at clinical stage II to IVA according to the 8th edition of the American Joint Committee on Cancer staging system (AJCC8) and an Eastern Cooperative Oncology Group (ECOG) performance status of 0 or 1 were consecutively included in a retrospective study conducted at four nationwide hospitals in China. Initially, all patients received PD-1 inhibitors (including pembrolizumab, toripalimab or tislelizumab) at standard approved doses, combined with chemotherapy (albumin-bound paclitaxel 260 mg/m^2^ on day 1 and nedaplatin 80 mg/m^2^ on day 1) administered every 3 weeks for 2 to 4 cycles. The inclusion criteria were patients who showed a poor radiological response—defined as stable disease (SD) or progressive disease (PD) based on Response Evaluation Criteria in Solid Tumors (RECIST) 1.1—and were still deemed eligible for surgical resection. Exclusion criteria included patients with distant metastatic disease at presentation, those who did not complete the prescribed treatment protocol, or those with evidence of esophageal perforation.

### Treatment protocol

For those patients, the secondary induction radiotherapy was administered in patients prior the surgery. All patients received intensity-modulated radiotherapy (IMRT) or tomotherapy targeting the primary tumor and clinically enlarged mediastinal lymph nodes. The median total dose delivered was 41.4 Gy (range, 39.6 to 45.0 Gy), with daily fractions of 1.8 to 2.0 Gy administered five times per week. Radiation dose constraints included a maximum spinal cord dose of less than 45 Gy, a mean lung dose below 15 Gy, and a lung V20 (volume receiving ≥20 Gy) less than 25%. For eligible patients undergoing radical esophagectomy, the McKeown procedure served as the standard surgical approach. Esophagectomy was typically scheduled 4 to 8 weeks following completion of neoadjuvant therapy. Pathological assessments were conducted by two senior pathologists in strict accordance with established protocols.

### Endpoints and assessments

The primary endpoint was the R0 resection rate, which was defined as the proportion of patients with no microscopic residual tumor at the resection margin. The secondary endpoints included the pathological complete response (pCR) rate, major pathological response (MPR) rate, objective response rate (ORR), disease control rate (DCR), tumor downstaging, and treatment-related safety outcomes. Tumor response was assessed by imaging and clinical examination in accordance with RECIST v1.1 at two timing points of post-nICT and post-secondary induction radiotherapy. However, a formal central imaging review was not performed with no re-reading. The ORR was defined as the proportion of patients who achieved complete or partial response; the DCR was defined as the proportion of patients who achieved complete response, partial response, or stable disease. Surgery was performed with curative intent following the completion of radiotherapy.

Pathological assessment of surgical samples was conducted to evaluate the treatment response. pCR was defined as the absence of viable tumor cells in the resected specimen. MPR was defined as a ≥90% reduction in the number of viable tumor cells. Tumor downstaging was determined by comparing the pretreatment clinical stage to the postoperative pathological stage; downstaging was defined as any reduction in tumor stage. The rates of pCR, MPR, R0 resection, and pathological downstaging were estimated using the Clopper–Pearson method, which provides exact binomial confidence intervals.

Safety outcomes included treatment-related adverse events (TRAEs), graded according to the National Cancer Institute Common Terminology Criteria for Adverse Events (NCI-CTCAE), version 5.0, and postoperative complications, which were graded via the Clavien–Dindo classification system.

### Statistical analysis

Descriptive statistics were used to summarize patient demographics, treatment responses, and surgical outcomes. The results are reported as absolute numbers, percentages, or medians, as appropriate. To assess survival outcomes, event-free (DFS) and overall survival (OS) probabilities at key time points were estimated using the Kaplan-Meier method and are reported with 95% confidence intervals. All the analyses were conducted via R software, version 4.3.

## Results

### Patient characteristics

**Figure 1:**
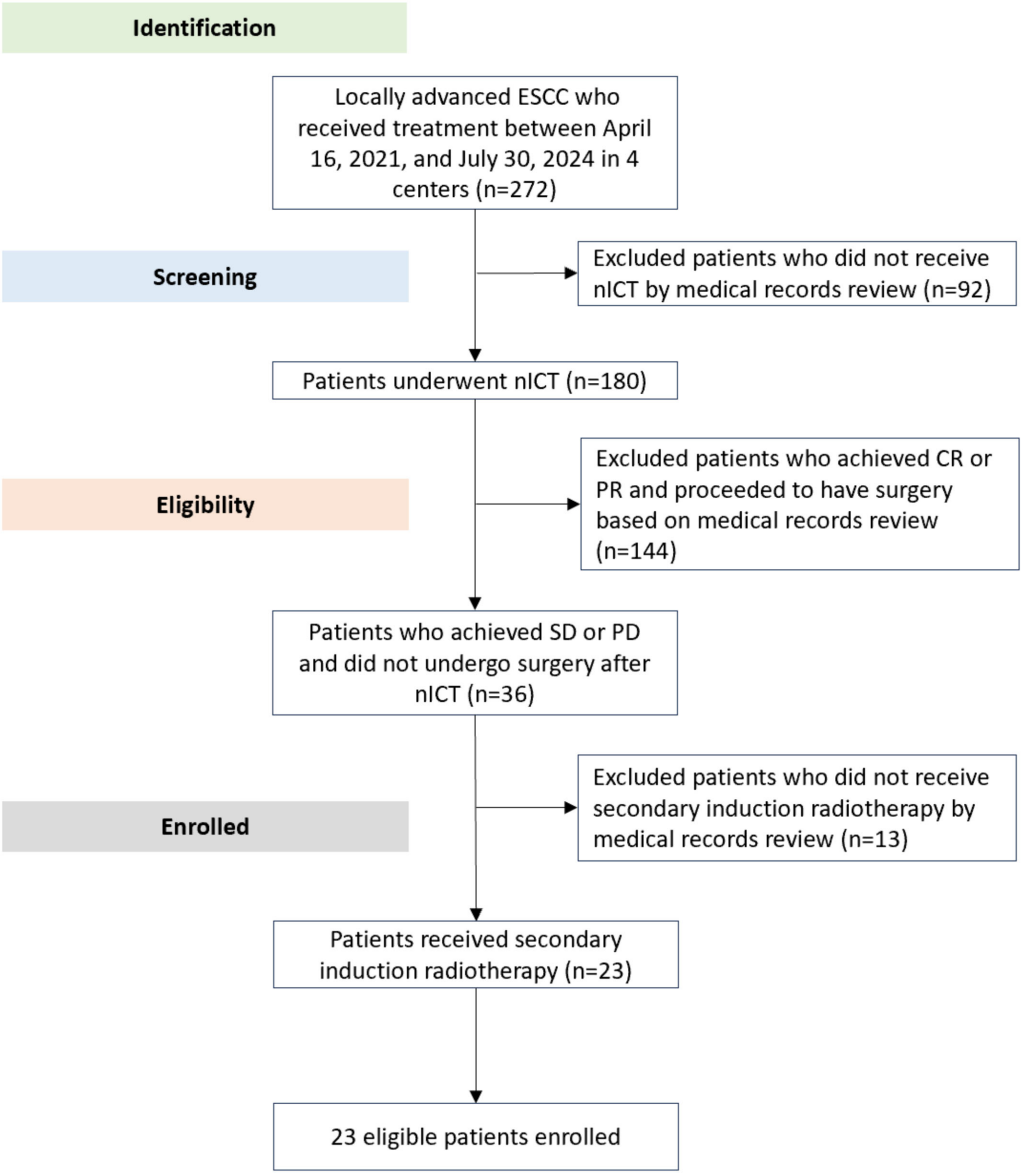
A retrospective study flowchart.

This retrospective study identified and enrolled 23 patients with locally advanced ESCC who received treatment between April 16, 2021, and July 30, 2024 (**Table 1**). The retrospective study flowchart depicting the consecutive enrollment process is shown in **Figure 1**. The median age was 63 years (range, 52-74), and 19 patients (82.6%) were male. Most patients (91.3%) had an ECOG performance status of 0. The tumor location was most commonly in the lower thoracic esophagus (43.5%), followed by the middle thoracic esophagus (39.1%). Following nICT, 30.4% of patients were classified as stage II, 60.9% as stage III, and 8.7% as stage IVA. Most patients (78.3%) had clinical T3 tumors at baseline. With respect to nodal status, 17.4% had cN0 disease, 47.8% had cN1 disease, 30.4% had cN2 disease, and 4.3% had cN3 disease. Radiographic assessment after nICT revealed stable disease in 19 patients (82.6%) and progressive disease in 4 patients (17.4%).

**Table 1:** Patient baseline characteristics.

Baseline characteristics	No. (%)
Age	
Median	63 (52-74)
Gender	
Male	19 (82.6)
Female	4 (17.4)
ECOG	
0	21 (91.3)
1	2 (8.7)
Tumor location	
Upper thoracic esophagus	4 (17.4)
Middle thoracic esophagus	9 (39.1)
Lower thoracic esophagus	10 (43.5)
Pathological type	
Squamous carcinoma	23 (100.0)
Clinical stage	
Stage II	7 (30.4)
Stage III	14 (60.9)
Stage IVa	2 (8.7)
T stage	
cT2	4 (17.4)
cT3	18(78.3)
cT4	1 (4.3)
N stage	
cN0	4 (17.4)
cN1	11 (47.8)
cN2	7 (30.4)
cN3	1 (4.3)
M stage	
cM0	23 (100.0)
Smoking	
Yes	17 (73.9)
No	6 (26.1)
Drinking	
Yes	14 (60.9)
No	9 (39.1)
Comorbidities	
Yes	11 (47.8)
No	12 (52.2)
Radiographic assessment after nICT	
SD	19 (82.6)
PD	4 (17.4)

SD, Stable disease; PD, Progressive disease.

### Treatment response

All 23 patients completed secondary induction radiotherapy. On post-radiotherapy imaging, 4 patients (17.4%) achieved a complete clinical response, 8 (34.8%) achieved a partial response, and 11 (47.8%) maintained stable disease, yielding an ORR of 52.2% and a DCR of 100% (**Table 2**). Pre- versus post-secondary induction radiotherapy, the dynamics of individual tumor size changes are depicted in **Supplementary Figure 1**. Pathological evaluation of the resected samples revealed MPR in 10 patients (43.5%) and pCR in 5 patients (21.7%) (**Table 3**). The impact of treatment cycles on outcomes was analyzed (**Supplementary Table 1**). Both the ≥3 cycles and <3 cycles groups achieved a 100% R0 resection rate. The MPR rate was numerically higher in the ≥3 cycles group (47.1% vs. 33.3%), but this difference was not statistically significant (p=0.66). These findings suggest that variation in nICT duration did not significantly confound the efficacy endpoints. Clinical downstaging was observed in 14 patients (60.9%), and pathological downstaging occurred in 16 patients (69.6%). At a median follow-up of 10 months (range, 3-31), disease recurrence was documented in 5 patients (21.7%), with 1 death during follow-up.

**Table 2:** Radiologic responses.

Clinical evaluation	No. (%)
CR	4 (17.4)
PR	8 (34.8)
SD	11 (47.8)
ORR	12 (52.2)
DCR	23 (100.0)

CR, Complete Response; PR, Partial response; SD, Stable disease; ORR, Objective Response Rate; DCR, Disease control rate.

**Table 3:** Pathologic responses.

Histological response	No. (%, 95% CI)*
MPR	10 (43.5, 23.2-65.5)
pCR	5 (21.7, 7.5-43.7)
Clinical Downstaging	14 (60.9, 38.5-80.3)
Pathological Downstaging	16 (69.6, 47.1-86.8)

MPR, Major pathologic response; pCR, Pathological complete response.

*95%CI were calculated based on the Clopper-Pearson method.

### Surgical outcomes

All 23 patients underwent radical resection with curative intent. The R0 resection rate was 100%. The median interval between the end of secondary induction radiotherapy and surgery was 48 days (range, 20-106). The median operative time was 310 minutes (range, 185-480), and the median intraoperative blood loss volume was 100 mL (range, 50-600). The median length of postoperative hospital stay was 10 days (range, 7-27). The surgical delay rate was 8.7% (2/23), and no ICU admissions occurred.

### Survival outcomes

**Figure 2:**
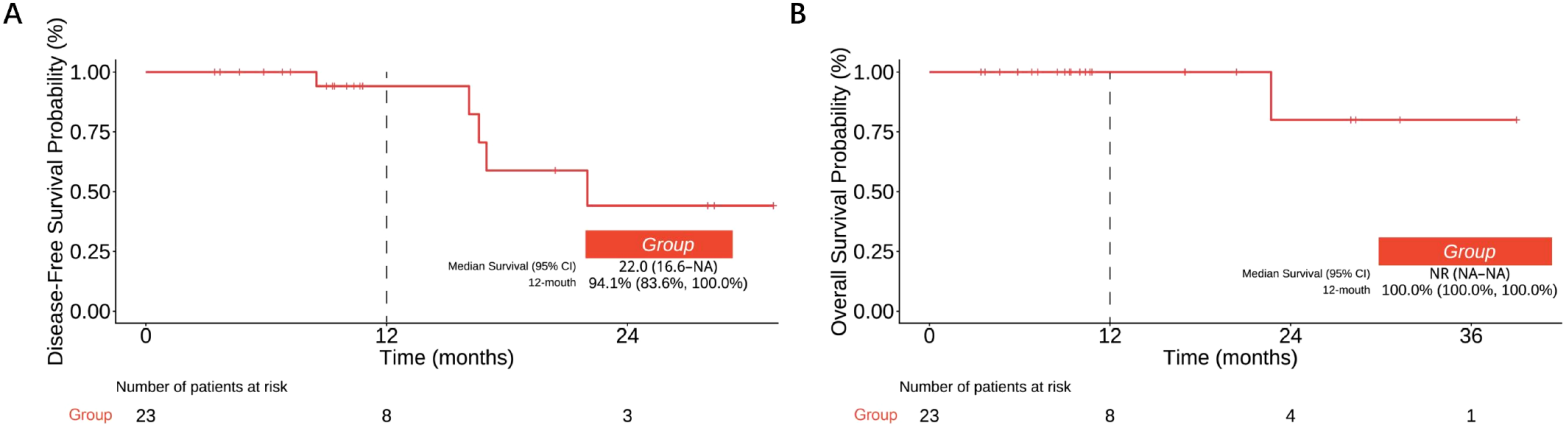
Survival outcomes: estimated DFS **(A)** and OS **(B)** rates (95% CI).

Median DFS was 22.0 months (95% CI 16.6-NA). The estimated 12-month DFS rate was 94.1% (95% CI 83.6%–100%) (**Figure 2A**). Median OS was not reached (NR) due to the limited number of events and the relatively short follow-up duration. The estimated 12-month OS rate was 100.0% (95% CI, 100.0%–100%) (**Figure 2B**). Disease recurrence occurred in 5 of 23 patients (21.7%) (**Supplementary Table 3**). Among the patients with recurrence, distant metastasis (DM) accounted for 60% (3/5) of the recurrence events, while locoregional recurrence (LR) constituted 40% (2/5). Specifically, 2 patients (8.7%) experienced locoregional recurrence, including one case of anastomotic recurrence and one case of mediastinal lymph node recurrence; 3 patients (13.0%) developed distant metastasis, involving the lung (n=1) and the liver (n=2).

### Safety

No intraoperative complications were reported. Postoperative complications occurred in 6 patients (26.1%), with grade 3–4 complications observed in 2 patients: pulmonary infection in 2 patients (8.7%) and anastomotic leakage in 1 patient (4.3%) (**Table 4**). The anastomotic leakage was managed with symptomatic and supportive care, anti-infective therapy, and enhanced nutritional support. Cases of pulmonary infection were treated with ventilator support combined with anti-infective therapy. All complications ultimately resolved. Perioperative complication rates were comparable between the high-dose and low-dose groups (33.3% vs. 21.4%; P = 0.643), suggesting that radiation dose level did not significantly influence surgical complication risk (**Supplementary Table 2**). A patient-level swimmer plot (**Supplementary Figure 2**) illustrating the exact interval (in days) between radiotherapy completion and surgery was analyzed with a median interval of 48 days. Treatment-related adverse events (TRAEs) related to radiotherapy occurred in 19 patients (82.6%). The grade 3–4 TRAEs included myelosuppression in 3 patients (13.0%) and gastrointestinal toxicity in 2 patients (8.7%) (**Table 5**). The myelosuppression events were treated with administration of granulocyte colony-stimulating factor (G-CSF). The gastrointestinal toxicities were managed by providing symptomatic supportive care along with corrective electrolyte supplementation. No treatment-related deaths occurred.

**Table 4:** Postoperative complications.

Event	Any Grade	Grade 1-2	Grade 3-4
Any events	6 (26.1%)	4 (17.4%)	2 (8.7%)
Anastomotic Leak	3 (13.0%)	2 (8.7%)	1 (4.3%)
Pulmonary Infection	3 (13.0%)	1 (4.3%)	2 (8.7%)
Arrhythmia	1 (4.3%)	1 (4.3%)	0

**Table 5:** Treatment-related adverse events.

Event	Any Grade	Grade 1-2	Grade 3-4
Any events	19 (82.6%)	13 (56.5%)	5 (21.7%)
Myelosuppression	12 (52.2%)	9 (39.1%)	3 (13.0%)
Gastrointestinal Reaction	7 (30.4%)	5 (21.7%)	2 (8.7%)
Thrombocytopenia	6 (26.1%)	6 (26.1%)	0
Dermatitis	5 (21.7%)	5 (21.7%)	0

## Discussion

To our knowledge, this retrospective study is the first research to evaluate the efficacy and safety of secondary induction radiotherapy in patients with locally advanced ESCC who demonstrated poor response to nICT. The findings suggested that the secondary induction radiotherapy yielded high rates of R0 resection, encouraging pathological responses and acceptable toxicity in this populations of patients, offering a potential therapeutic regimen to curative surgery for this challenging subgroup.

Immunochemotherapy has emerged as a key component of neoadjuvant treatment for ESCC. In the phase III ESCORT-NEO/NCCES01 trial, patients receiving camrelizumab combined with chemotherapy achieved significantly higher pCR and MPR rates than those receiving chemotherapy alone did (28.0% and 59.1% in Group A vs. 4.7% and 20.9% in Group C, respectively) (26). As reported in the ESONICT-1 study, neoadjuvant sintilimab combined with chemotherapy achieved a pCR rate of 21.7% and a MPR rate of 52.2% (18). Similarly, the ESONICT-2 trial demonstrated that the regimen of toripalimab plus docetaxel and cisplatin yielded a pCR of 16.7% and a MPR of 41.7% (21). These findings are consistent with the broader landscape of phase II trials in this setting, which have reported pCR rates ranging from 20% to 50%, along with encouraging tumor downstaging and surgical feasibility (15–17). However, a proportion of patients showed limited tumor regression following nICT (27). These nICT-insensitive patients are considered unlikely to achieve a favorable pathological response or long-term survival after surgery. In a prospective phase II trial (ChiCTR2100045722) enrolling locally advanced ESCC patients who received nICT, the pCR rates in the responder (CR/PR) and non-responder (SD/PD) groups were 32.2% and 0.0%, respectively. Compared with the non-responder group, the responder group trended toward prolonged DFS (p=0.310) and OS (p=0.08) (28). A real-world retrospective study further showed that the 2-year DFS rate in locally advanced ESCC patients with CR/PR following nICT was significantly higher than that in patients with SD/PD (83.2% vs. 59.8%, P = 0.04), with a similar trend observed for the 2-year OS rate (89% vs. 63.8%, P = 0.02) (29). Therefore, the treatment strategy proposed in this study, which involves introducing a second induction radiotherapy to further improve clinical response rates in ESCC patients with SD/PD after nICT, is of great necessity. In our study, all 23 patients who showed poor response to nICT completed secondary induction radiotherapy prior surgery. The efficacy was assessed as CR (17.4%) in 4 patients, PR (34.8%) in 8 patients, and an ORR of 52.2% and a DCR of 100%.

Radiotherapy has long been recognized for its capacity to induce tumor shrinkage and improve resectability (22–25). In our study, 23 patients with a poor response to nICT underwent secondary induction radiotherapy. More than half (52.2%) of patients achieved an objective response, and all of them proceeded to surgery, with an R0 resection rate of 100%. Postoperative histopathological analysis revealed a pCR rate of 21.7% and an MPR rate of 43.5%. These results were comparable to historical data on nICT alone in ESCC (4, 15–18, 21, 26, 30) and suggested that radiotherapy represented a trend of restoring treatment sensitivity and surgical eligibility in this refractory cohort. Disease recurrence occurred in 5 patients, with 1 death during follow-up. Although long-term survival outcomes still needed support by further follow-up data, the universal achievement of R0 resection which was an established prognostic factor in esophageal cancer was encouraging (31). In one retrospective study, patients who underwent R0 resection had significantly better survival outcomes than those who underwent noncurative (R1 or R2) resection (P < 0.0001) (32).

The optimal radiation dose for neoadjuvant treatment of esophageal cancer remains debated. A number of retrospective studies have evaluated the impact of dose intensity, but the results have been inconsistent (33–37). A trial of 141 patients with stage III ESCC reported that a higher dose (50–50.4 Gy) was associated with improved pCR and 3-year survival compared with a lower dose (36 Gy) (31). However, a meta-analysis using biologically effective dose (BED) stratification reported superior survival and lower toxicity in the low-dose group (BED ≤48.85 Gy) (33). Subgroup analyses further favored the 40-41.4 Gy dose range, which was associated with improved progression-free survival (PFS) and overall survival (OS) and lower rates of distant metastasis. Our findings supported the use of 41.4 Gy as the promising dose for secondary induction radiotherapy in ESCC patients who demonstrated poor response to nICT. This regimen was associated with high rates of resection and pathological response while maintaining an acceptable safety profile. The events of postoperative complication were rare, with only 2 patients (8.7%) experiencing grade 3–4 surgical complications. Radiation-induced toxicity was also manageable, with grade 3–4 TRAEs, including myelosuppression (13.0%) and gastrointestinal symptoms (8.7%). A safety comparison with the nCRT group from the NEOCRTEC5010 trial revealed a markedly lower incidence of severe myelosuppression in our cohort (13.0% vs. 48.9%, P < 0.001) (4). In contrast, the rate of severe gastrointestinal toxicity did not differ significantly from the historical benchmark (8.7% vs. 4.0%, P = 0.274). Collectively, these data suggest an accepted safety tolerance for our treatment strategy of adding secondary induction radiotherapy for ESCC patients with a poor response to nICT.

Regarding long-term survival and recurrence patterns, our study found that incorporating secondary induction radiotherapy did not change the recurrence profile after neoadjuvant immunochemotherapy. The distribution of first recurrence sites remained similar, with distant metastases continuing to predominate over locoregional failure. The spectrum of involved metastatic sites was also comparable to that reported in other study (38).

This study has several limitations. Owing to its retrospective design and sample size, exemplified by the wide confidence interval for the pCR rate (21.7%, 95% CI 7.5%–43.7%), potential for selection bias should be acknowledged, and broader generalizability of the findings may require validation in larger cohorts. The lack of a comparative group limits direct inference on treatment efficacy, though the study underscores the feasibility of secondary induction radiotherapy in this specific patient population. Without a direct comparator cohort (e.g., patients undergoing surgery alone or continuing with nICT alone), the observed clinical benefits cannot be definitively isolated from potential selection biases. Consequently, the outcomes may partly reflect a “pseudo-benefit”, attributable to the enrollment of patients with favorable baseline biology or those already in a state of disease control after prior therapy, rather than solely to the therapeutic effect of radiotherapy. These observations highlight the need for prospective investigations to further characterize the role of such multi-modal approaches in clinical practice. Furthermore, the follow-up duration was insufficient to assess long-term survival outcomes. Given the study’s limitations, including susceptibility to selection bias, small sample size uncertainty, and a short follow-up that precludes assessment of long-term survival benefit, the observed promising pathological responses should be interpreted with caution. Although the observed R0, pCR and MPR rates in our cohort appeared to fall within the spectrum of rates reported in prior prospective trials of nICT alone, these findings must be regarded as preliminary and require validation in larger, prospective studies whether these favorable responses translate into long-term survival benefit.

Our work conducted an initial, hypothesis-generating assessment in this challenging patient population (poor responders to nICT). Therefore, building on these preliminary findings, future research should aim to optimize the integration of immunotherapy, chemotherapy, and radiotherapy in a sequential or concurrent fashion by using a controlled study design. Further efforts are needed to refine treatment sequencing, identify biomarkers predictive of response, and personalize treatment on the basis of tumor biology. These strategies are critical for improving long-term outcomes and quality of life in patients with locally advanced ESCC.

## Conclusion

This study provides preliminary evidence that secondary induction radiotherapy is a feasible and effective approach for patients with locally advanced ESCC who fail to respond to nICT. The high R0 resection rate, encouraging pathological response, and manageable toxicity observed in this cohort support further investigation of this strategy in prospective clinical trials.

## Data Availability

The original contributions presented in the study are included in the article/**Supplementary Material**. Further inquiries can be directed to the corresponding author.
