# Supplementary material for: Efficacy and safety of secondary induction radiotherapy in locally advanced resectable esophageal squamous cell carcinoma with poor responses to neoadjuvant immunochemotherapy: a retrospective study
Source: Front Immunol. 2026 Mar 11;17:1656631. doi: 10.3389/fimmu.2026.1656631 (PMC13013412; doi:10.3389/fimmu.2026.1656631)
Supplement: Supplementary file 1 [file Table1.docx]

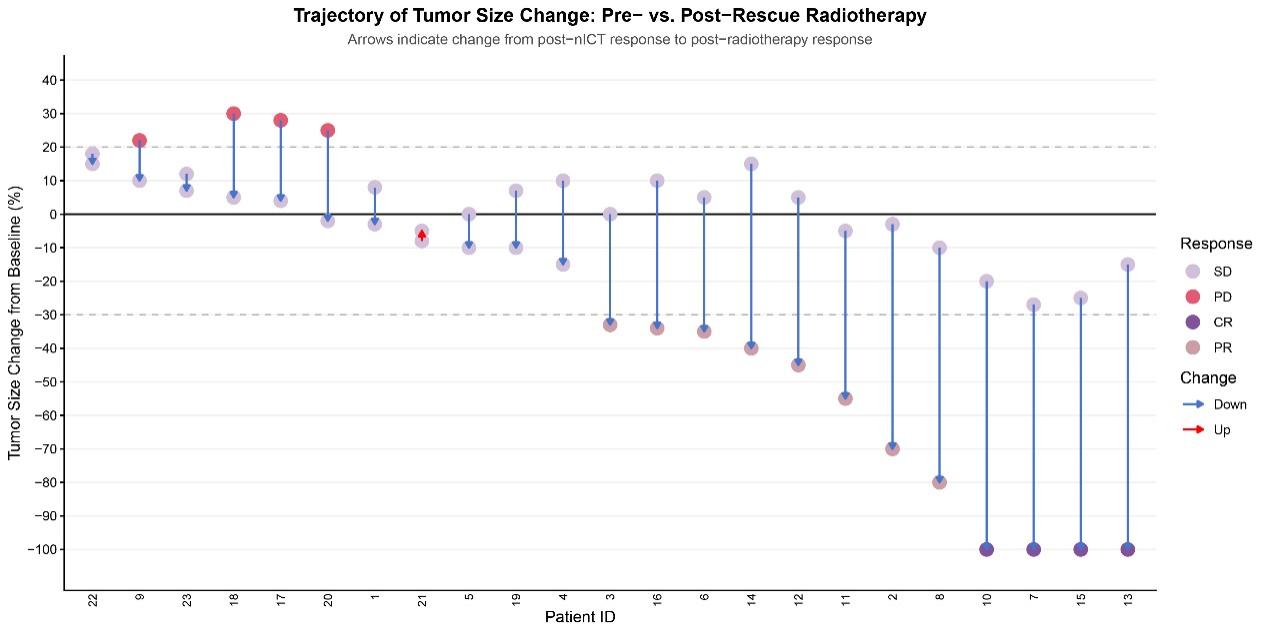


**Supplementary Figure 1:** Trajectory of Tumor Size Change: Pre-vs. Post-secondary induction radiotherapy.

Note: Arrows indicate change from post-nlCT response to post-radiotherapy response. **Arrow colors:** Up (red) indicates deterioration, meaning the tumor size increased after secondary induction radiotherapy; Down (blue) indicates improvement, meaning the tumor size decreased after secondary induction radiotherapy; No (gray) indicates no change, meaning the tumor size remained the same after secondary induction radiotherapy. **Arrow direction:** The starting point represents the tumor size after initial neoadjuvant chemotherapy, and the arrowhead represents the tumor size after secondary induction radiotherapy.


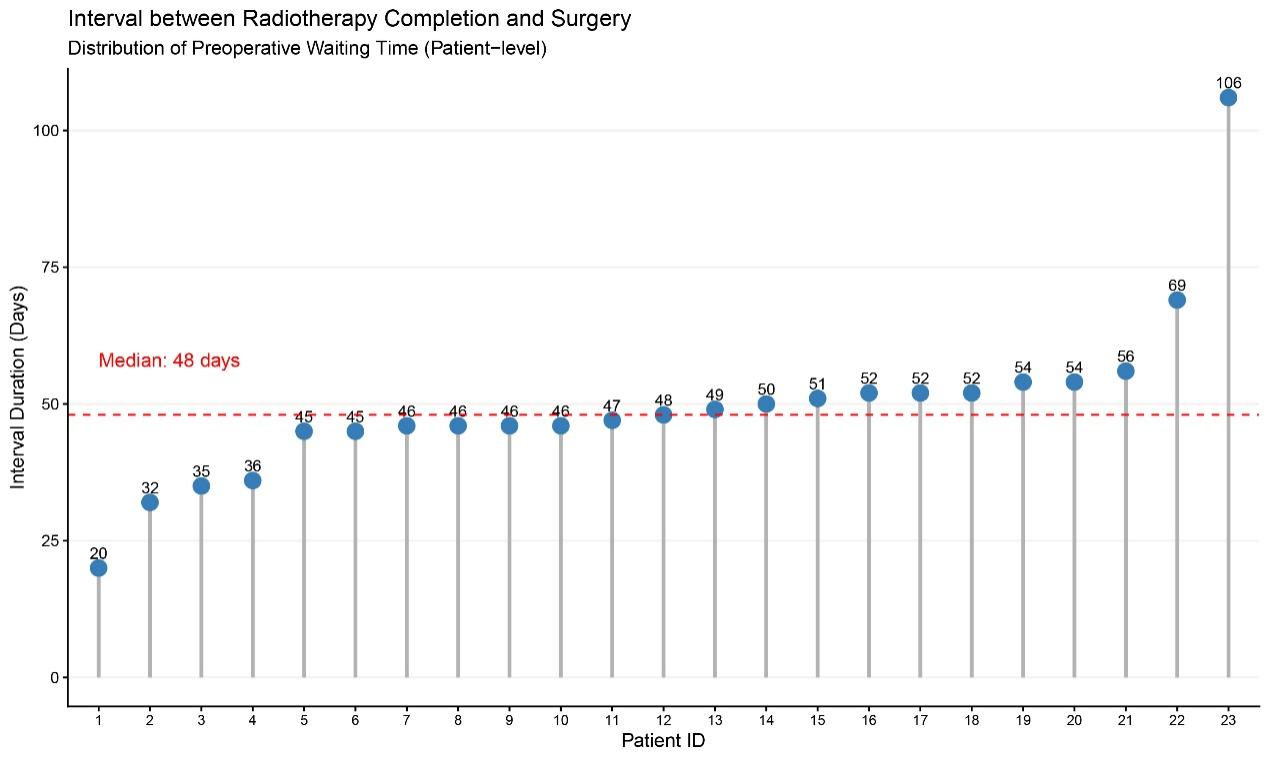


**Supplementary Figure 2:** Interval between Radiotherapy Completion and Surgery Distribution of Preoperative Waiting Time (Patient-level).

**Supplementary Table 1:** Comparison of MPR and R0 Resection Rates: Low-Cycle (<3) vs. High-Cycle (≥3) nICT Groups

|  |  | Low cycles  （N=6） | High cycles  （N=17） | P value |
| --- | --- | --- | --- | --- |
| MPR | Yes | 2 (33.3%) | 8 (47.1%) | 0.660 |
|  | No | 4 (66.7%) | 9 (52.9%) |  |
| R0 resection | Yes | 6（100%） | 17（100%） | 1.000 |
|  | No | 0 | 0 |  |

**Supplementary Table 2:** Perioperative Indicators and Complication Rates by Radiation Dose Group

|  |  | High Dose  （N=9） | Low Dose  （N=14） | P value |
| --- | --- | --- | --- | --- |
| Complications | Yes | 3 (33.3%) | 3 (21.4%) | 0.643 |
|  | No | 6 (66.7%) | 11 (78.6%) |  |
| Age |  | 64.8 (6.9) | 63.3 (6.1) | 0.595 |
| Operation time (min) |  | 280 (46.2) | 334 (82.5) | 0.092 |
| Blood loss (ml) |  | 103 (49.2) | 204 (173) | 0.309 |
